# Extended Anionic Network in Mixed‐Valent Nitridocobaltates(I/II) *Ln*Co_2_N_2_ (*Ln* = La, Pr, Nd)

**DOI:** 10.1002/advs.76531

**Published:** 2026-07-09

**Authors:** Nina A. M. Prinz, Jonas M. Albrecht, Dominik Werhahn, Simon Steinberg, Clemens Ritter, Simon D. Kloß

**Affiliations:** ^1^ Department Chemistry LMU Munich Munich Germany; ^2^ Institute of Inorganic Chemistry RWTH Aachen University Aachen Germany; ^3^ Institut Laue‐Langevin Grenoble France

**Keywords:** high‐pressure synthesis, neutron diffraction, nitrides, transition metals

## Abstract

Nitridometallates with extended anionic frameworks exhibit a variety of intriguing electronic properties, including band magnetism, metal‐metal bonding, and superconductivity. Such extended framework materials are scarce with late transition metals due to the requirement of high metal oxidation states and nitrogen content. This study presents a family of nitridocobaltates *Ln*Co_2_N_2_ (*Ln* = La, Pr, Nd) with an extended layered cobalt‐nitrogen network related to the Kagome lattice that was synthesized at 8 GPa in a large volume press. The compounds crystallize in the trigonal space group *R* $\bar{3}$ with alternating layers of unusual trigonal planar CoN_3_ and octahedral *Ln*N_6_ polyhedra. Magnetization and powder neutron diffraction studies indicate a metallic ground state and the suppression of magnetic ordering of the rare earth moments due to their arrangement on a trigonal lattice. The compounds have a surprising mixed‐valent Co^+I/+II^ state, which is high for nitridocobaltates. This study demonstrates the structural diversity available in late nitridometallates via high‐pressure synthesis, which can now be explored systematically.

## Graphical abstract

This study presents the high‐pressure synthesis of *Ln*Co_2_N_2_ (*Ln* = La, Pr, Nd) with Co in mixed valent states of +I/+II. The compounds demonstrate the stabilization of a nitridocobaltate with extended anionic Co–N layers where Co is threefold coordinated.

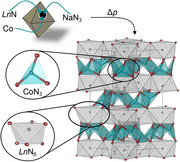

## 1 Introduction

It is well known that electropositive cations such as alkaline earths or rare earths stabilize transition metal nitrides with nitrogen‐rich compositions and high oxidation states compared to their parent binary nitrides [1, 2]. This premise, usually rationalized with the inductive effect of electropositive cations, was demonstrated by numerous examples of nitridometallates A_x_M_y_N_z_ with high *A*:*M* ratios, such as the families of *AE*_5_ *M*N_5_, *AE*_3_ *M*N_3_, *AE*_2_ *M*N_2_ materials where *AE* is an alkaline earth [3, 4]. A caveat is, however, that while high *A*:*M* ratios can lead to high oxidation states, they usually result in low dimensional nitridometallate anions such as isolated trigonal planar *M*N_3_ or tetrahedral *M*N_4_ moieties [5]. Decreasing the *A*:*M* ratio can result in anionic networks extended in two or three dimensions but are much rarer and currently only known for early transition metals up to Mn [6, 7]. In oxides, extended framework materials with low *A*:*M* ratios are well investigated and have commercial applications stemming from strongly correlated electron physics such as the (Ba,Sr)Fe_12_O_19_ ferrites or spinel‐type materials AB_2_O_4_ used as permanent and soft magnets, respectively [8, 9]. Being able to tune the *A*:*M* ratio gives control over the anion network topology, which can be crucial as the commercial success of nitridosilicate, ‐aluminate, and ‐phosphate luminescent materials demonstrated [10, 11, 12]. Development of a systematic access to nitridometallates, especially to extended frameworks of late transition metal nitrides, could lead to discovery of useful quantum materials in analogy to oxides.

The presently known nitridometallates with extended frameworks display curious electronic ground states such as charge‐transfer metallic behavior in Li_2_Sr[MnN]_2_ containing 1D linear chains of [MnN_2/2_], semiconducting Ca_12_[Mn_19_N_23_] and Ca_133_[Mn_216_N_260_] with 2D layered structures and metal–metal bonding, and superconducting *Ln*_3_Cr_10–_ *_x_* N_11_ (*Ln* = La, Pr) with a 3D framework of CrN_4_ tetrahedra [6, 13, 14, 15]. Similar frameworks are not known for transition metals from iron to copper except for a subnitridonickelate Ba_2_[Ni_3_N_2_] with mean Ni oxidation states of +0.67 [16]. Otherwise, a *A*:*M* ratio of one represents the minimum as realized in the *AEM* ^I^N family of materials with *AE* = Ca, Sr, Ba and *M* ^I^ = Co^I^, Ni^I^, Cu^I^ that feature 1D chains of *M*N_2_ linear dumbbells [17, 18, 19, 20, 21]. The simultaneous stabilization of extended networks and higher oxidation states is thus difficult for late transition metals. With cobalt, the highest oxidation state in nitridocobaltates was limited to +I and coordination numbers to two, the latter realized either as zero‐dimensional CoN_2_ dumbbells like in Ca_5_[CoN_2_]_2_ and LiSr_2_[CoN_2_] or as one‐dimensional chains like in Ba[CoN] [22, 23, 24].

High‐throughput computations showed that the ternary nitrides of cobalt are less stable than for the early transition metals, making their synthesis challenging [25, 26]. High‐pressure synthesis offers highly oxidizing conditions, as demonstrated in the preparation of transition metal nitrides such as Ca_4_Fe^IV^N_4_, Ca_2_Ni^II^N_2_, as well as nitride perovskites and Ruddlesden‐Popper nitrides [5, 27, 28, 29, 30]. Here, we prepare a family of materials *Ln*Co_2_N_2_ (*Ln* = La, Pr, Nd) with a ratio *A*:*M* of 1:2 by high‐pressure synthesis. The materials have a 2D layered anionic network, which is topologically related to the famous Kagome lattice, consisting of unusual trigonal planar CoN_3_ moieties with mixed‐valent Co^+I/+II^ states. Neutron diffraction experiments reveal full nitridation of the compounds, while magnetization measurements and computations based on density functional theory indicate a surprising metallic ground state, which places the materials on the border of intermetallics and classic nitridometallates.

## 2 Results and Discussion

### 2.1 Synthesis and Chemical Analysis

The *Ln*Co_2_N_2_ (*Ln* = La, Pr, Nd) materials were prepared according to Equation 1 at 8 GPa and 850°C in a multianvil large‐volume press and obtained as a black microcrystalline powder with crystallite sizes of up to 15 µm and ca. 40 mg yield per experiment (Figure S1).

(1) $$\mathrm{Ln}N+2\;\mathrm{Co}+2\;\mathrm{Na}N_{3}\;\to \mathrm{Ln}{\mathrm{Co}}_{2}N_{2}+2\;\mathrm{Na}+2.5N_{2}$$

A 10% excess of Co and NaN_3_ was needed to optimize the formation of the *Ln*Co_2_N_2_ materials, while small amounts of *Ln*N remained as a byproduct. Additionally, a non‐identifiable byproduct, consistent across all compounds, was identified in the neutron diffraction patterns (Figure 2). In contrast to literature reports on nitridocobaltates (e.g. *AE*_5_(CoN_2_)_2_, where *AE* = Ca, Sr), the crystals of *Ln*Co_2_N_2_ (*Ln* = La, Pr, Nd) are resistant to air for several months as well as short exposures to diluted hydrochloric acid, but show signs of surface hydrolysis or passivation as found by scanning electron microscopy (Figure S1) [31].

Despite surface passivation, energy dispersive X‐ray (EDX) spectroscopy on samples washed with diluted hydrochloric acid (0.1 M) (normalized on *Ln*, 7–10 datapoints averaged, Table S1) revealed chemical formulas of La_1.0(1)_Co_2.1(3)_N_1.6(4)_, Pr_1.0(2)_Co_2.2(2)_N_2.1(3)_, and Nd_1.0(1)_Co_2.0(2)_N_2.1(3)_, which are close to the expected compositions. The composition of the compound was further confirmed by powder neutron diffraction experiments carried out on all *Ln*Co_2_N_2_ samples at the D20 high‐intensity two‐axis neutron diffractometer of the Institut Laue‐Langevin, which indicated full nitrogen position occupancies after tentative Rietveld refinement (details in a later section).

### 2.2 Structure Discussion

Single‐crystal X‐ray diffraction data were collected for all *Ln*Co_2_N_2_ materials at ambient temperature. Systematic missing reflections, Laue symmetry, and cell metrics of all three materials are consistent with trigonal space group *R* $\bar{3}$ (no. 148) and lattice parameters of *a* = 6.5664(6) Å and *c* = 15.568(2) Å for LaCo_2_N_2_, *a* = 6.4955(5) Å and *c* = 15.3677(16) Å for PrCo_2_N_2_, and *a* = 6.4702(3) Å and *c* = 15.3123(16) Å for NdCo_2_N_2_. The change in lattice parameters and volumes (*V*_La_ = 581.31(13) Å^3^, *V*_Pr_ = 561.52(10) Å^3^, *V*_Nd_ = 555.14(8) Å^3^) follow the expected trend owing to the lanthanoid contraction [32] and is in line with the shannon radii of the lanthanides for a coordination number of *CN* = 6 (La: 1.172 Å, Pr: 1.13 Å, Nd: 1.123 Å) [33].

Initial structure solution via intrinsic phasing resulted in the positions of the heavy atoms, while nitrogen positions were determined from the difference Fourier map [34]. All atoms were refined with anisotropic displacement parameters, and the lists of atom positions are shown in Table 1. The crystallographic data, displacement parameters, and interatomic distances and angles are summarized in Tables S2–S8.

**TABLE 1:** Atomic coordinates and equivalent anisotropic atomic displacement parameters for *Ln*Co_2_N_2_ (*Ln* = La, Pr, Nd). Standard deviations are given in brackets.

Atom	*x*	*y*	*z*	Occ.	*U*_eq_ [Å^2^]	wyckoff	Symm.
**LaCo_2_N_2_**							
La1	0	0	0	1	0.00516(17)	3*a*	−3.
La2	0	0	0.36638(3)	1	0.00446(15)	6*c*	3.
Co1	0.03007(10)	0.24006(10)	0.18522(4)	1	0.00453(16)	18*f*	1
N1	0.3824(6)	0.0766(7)	0.0841(2)	1	0.0062(7)	18*f*	1
**PrCo_2_N_2_**							
Pr1	0	0	0	1	0.0053(2)	3*a*	−3.
Pr2	0	0	0.36542(4)	1	0.0043(2)	6*c*	3.
Co1	0.03169(15)	0.24208(15)	0.18556(6)	1	0.0049(2)	18*f*	1
N1	0.3803(9)	0.0759(9)	0.0823(3)	1	0.0058(11)	18*f*	1
**NdCo_2_N_2_**							
Nd1	0	0	0	1	0.00485(10)	3*a*	−3.
Nd2	0	0	0.36521(2)	1	0.00461(8)	6*c*	3.
Co1	0.03236(8)	0.24264(8)	0.18559(3)	1	0.00409(10)	18*f*	1
N1	0.3789(5)	0.0751(5)	0.08148(18)	1	0.0049(5)	18*f*	1

**FIGURE 1:**
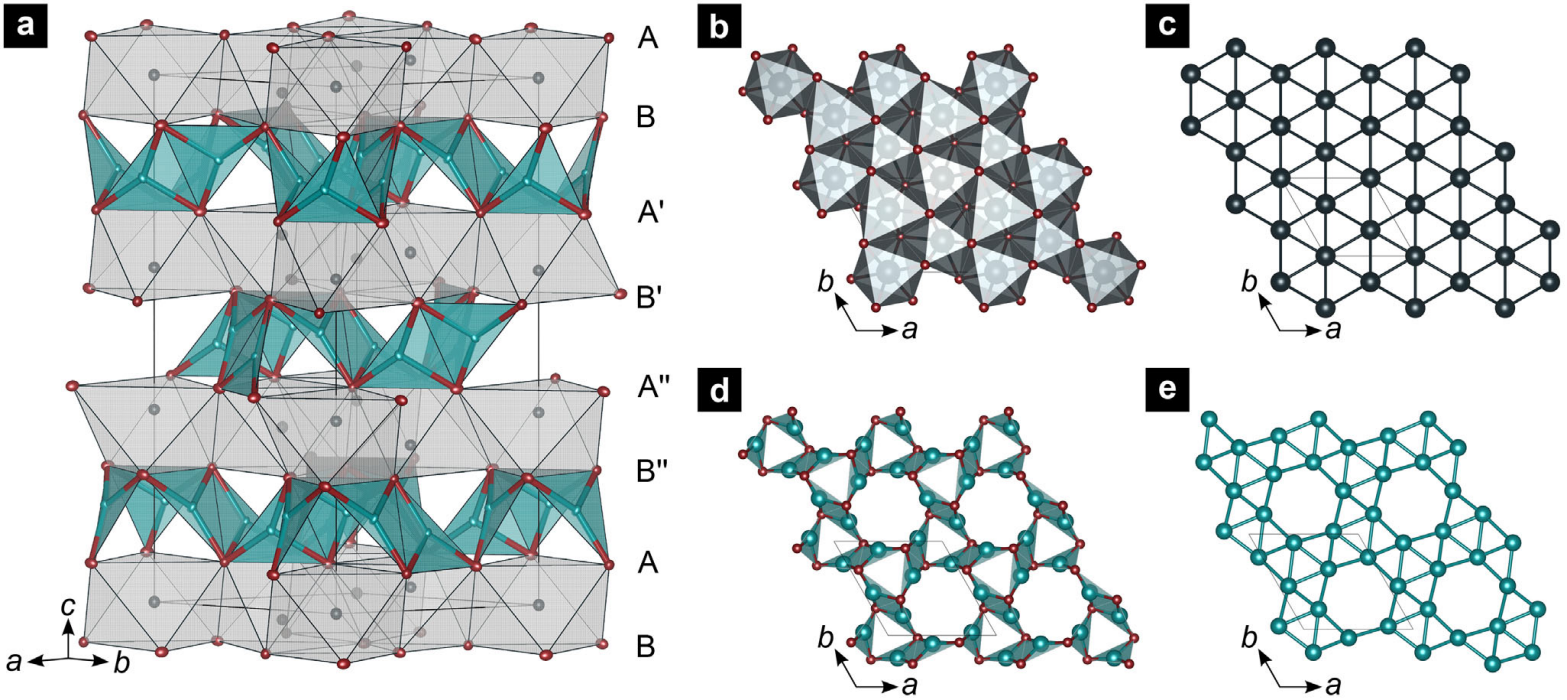
(a) Layered structure of *Ln*Co_2_N_2_ with *Ln*N_6_ (black) and CoN_3_ (blue) polyhedra. Nitrogen is colored red. ABA'B′A″′B″′ denotes the distorted hexagonal AB packing of the nitride anions. The atoms are shown as displacement ellipsoids with a probability of 90%. (b) *Ln*N_6_ network and (c) network topology of the Nd‐atoms in projection along [001]. (d) CoN_3_ network and (e) Co network topology in projection along [001].

The *Ln*Co_2_N_2_ (*Ln* = La, Pr, Nd) compounds crystallize (Figure 1a) in an unprecedented structure type consisting of distorted hexagonal AB packing of nitride anions, where *Ln* and Co occupy voids in alternating layers (Figure 1b, d) that are stacked along the *c* direction. In these materials, Co exhibits an, for nitridocobaltates, unprecedented mixed valent state of +I/+II, and the *Ln*Co_2_N_2_ materials therefore give precedence for the stabilization of Co^+II^ in nitridocobaltates. Bond valence sum (BVS) calculations were performed to further corroborate these oxidation states but remained inconclusive due to the unprecedented bonding situation in the *Ln*Co_2_N_2_ compounds (details of BVS in the Supporting Information). Therefore, electronic structure analysis based on computational means is discussed in a later section.

The *Ln*N layers have *Ln* in two distinct sites with distorted octahedral *Ln*N_6_ coordination (Figure 1b), while in the CoN‐layers, Co occupies distorted trigonal‐planar voids (Figure 1d). Previously, only linear coordination has been reported in nitridocobaltates, while trigonal‐planar moieties are known only for less electronegative metals Cr, Mn, and Fe, as in Ca_3_CrN_3_, Ca_12_Mn_19_N_23_ and Ba_3_FeN_3_ [6, 35, 36]. For cobalt, such a motif was reported only for complexes with amido ligands such as Co[N(SiMe_3_)_2_]_3_ and the binary interstitial nitride Co_2_N [37, 38]. The occupation of trigonal‐planar voids in closest packings is unusual in general, but was, for example, reported for deuterium in compounds such as SmD_3_ or SmMg_2_D_7_ [39].

The CoN_3_ units that themselves are interconnected via common corners and edges; the resulting network forms 3‐rings and 6‐rings within the *ab*‐plane according to the nomenclature introduced by Liebau for tetrahedral networks [40]. A graph theoretical description of the network topology was calculated with the program TOPOS Pro; it can be described as a Shubnikov plane net with vertex symbol 3^4^.6, which develops a uniform, Archimedean network (Figure 1e) [41]. This network is related to the famous Kagome‐type lattice, with vertex symbol 3.6.3.6, via extension of the spacing between 6‐rings by additional 3‐rings.

Three different Co–Co distance (La‐phase: 2.3341(11) Å, 2.5628(7) Å and 2.5763(11) Å; Pr‐phase: 2.3265(17) Å, 2.5256(10) Å and 2.5638(15) Å; Nd‐phase: 2.3245(9) Å, 2.5124(5) Å and 2.5575(8) Å) were found within this network, the shortest distances representing the Co–Co spacing of two edge‐sharing CoN_3_ polyhedra. These distances are in the same range or shorter than the Co–Co distance in cubic Co metal (2.50 Å) and indicate Co–Co bonding, which is discussed in the later DFT section [42].

### 2.3 Powder Neutron and X‐Ray Diffraction

**FIGURE 2:**
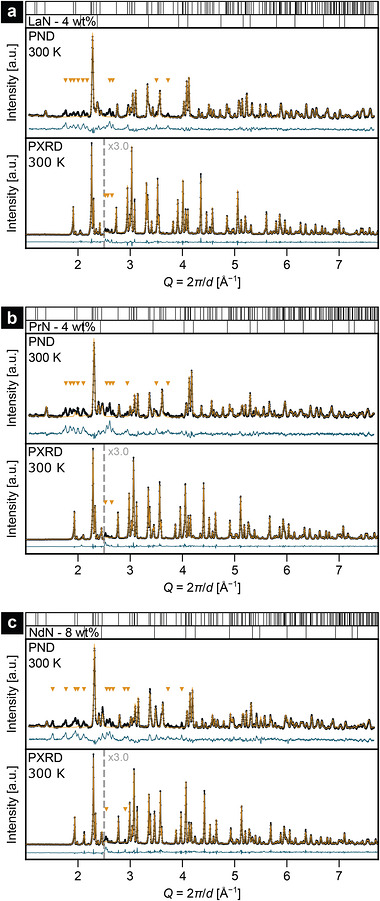
In‐house X‐ray and neutron co‐refinement of (a) LaCo_2_N_2_, (b) PrCo_2_N_2_ and (c) NdCo_2_N_2_ with powder data obtained at 300 K. Black lines indicate the observed data, orange lines the Rietveld fits, and the blue lines the difference plots. Theoretical reflection positions of *Ln*Co_2_N_2_ and *Ln*N are marked above in black. Unidentified reflections are marked by orange triangles. For clarity, the PXRD data are shown between Q  =  1 – 7.75 Å^−1^ while a blowup of the full diffractograms is shown in Figure S2. Furthermore, the regions with Q > 2.5 Å^−1^ are magnified x3.0 (indicated by a grey dashed line).

The in‐house X‐ray diffractometer provided good resolution for the heavy atoms *Ln* and Co but is insensitive toward N, whereas the neutron data have a lower resolution for *Ln* and Co but give excellent sensitivity to the nitrogen atom. Therefore, the crystal structure was co‐refined against both datasets simultaneously to confirm the single‐crystal structure determination. The initial refinement of the data confirmed that *Ln*Co_2_N_2_ (*Ln* = La, Pr, Nd) are isotypic and crystallize in the trigonal space group *R* $\bar{3}$ (no. 148). Rietveld fits (Figure 2) were performed for data obtained at 300 K: The lattice parameters determined were *a*  =  6.57185(8) Å and *c * =  15.5650(3) Å for LaCo_2_N_2_, *a*  =  6.49992(6) Å and *c * =  15.3644(2) Å for PrCo_2_N_2_ and *a * =  6.47769(17) Å and *c * =  15.3081(4) Å for NdCo_2_N_2_, which is in good agreement with the data obtained from SCXRD data. The crystallographic data of the powder refinement are summarized in Tables S10–S18. Since powder neutron diffraction is highly sensitive to nitrogen atoms, refinements of the nitrogen site occupancies based on the powder neutron diffraction data gave 107(2)%, 100(3)%, and 103(3)% N occupancy for La‐phase, Pr‐phase, and Nd‐phase, respectively, indicating that oxygen impurities or nitrogen vacancies are either minor or not present. All phases exhibited a similar unidentified impurity, which was only visible in the neutron data and had not been detected in the previous in‐house powder X‐ray diffraction analyses. This could be attributed to an impurity phase containing light elements. Furthermore, binning of several samples was required to acquire enough sample for the neutron measurements.

### 2.4 Magnetometry

Magnetization measurements were performed for all three *Ln*Co_2_N_2_ (*Ln* = La, Pr, Nd) compounds to obtain the temperature‐dependent susceptibility in fields of 100 Oe and 30 kOe in the range of 2 to 300 K (400 K in the case of NdCo_2_N_2_), while field‐dependent measurements were conducted at 2 and 300 K in the range of ± 50 kOe.

#### 2.4.1 LaCo_2_N_2_

Susceptibility measurements on LaCo_2_N_2_ showed a low susceptibility with Curie‐type behavior at high temperatures. A Curie‐type fit as $X_{mol}=X_{const}+\frac{C}{T}$ in the range from 200 to 300 K, including a refined constant magnetic moment of *Χ*_const_ = 2.34·10^−4^ cm^3^·mol^−1^ yielded an effective moment of *µ*_eff_ = 0.77(1) µ_B_ per Co atom. This is well below the spin‐only value expected from Co^+I/II^ in trigonal planar coordination, leading to *S* = ½ and *S* = 1 configurations with $\mu_{eff}\;per\;Co=\;\sqrt{\frac{\mu_{eff\_+I}^{2}+\mu_{eff\_+II}^{2}}{2}}=2.34\;\mu_{B}.$ This indicates that the magnetic moment of cobalt is quenched, which probably is due to metal–metal bonding and a metallic ground state as discussed in the DFT‐section. The low effective magnetic moment may stem from partly localized electron behaviour but more likely stems from impurities, e.g., 4% of a *S* = 3/2 impurity, which represents Co^2+^ in an octahedral ligand field. The powder neutron diffraction pattern detected a nitrogen‐rich impurity on the ∼10% level that may be responsible for this magnetic signal. The field‐dependent measurements at 2 K also show small amounts of a ferromagnetic impurity and an overall small magnetic moment of ca. 0.035 µ_B_ at 50 kOe (Figure S3).

#### 2.4.2 PrCo_2_N_2_

**FIGURE 3:**
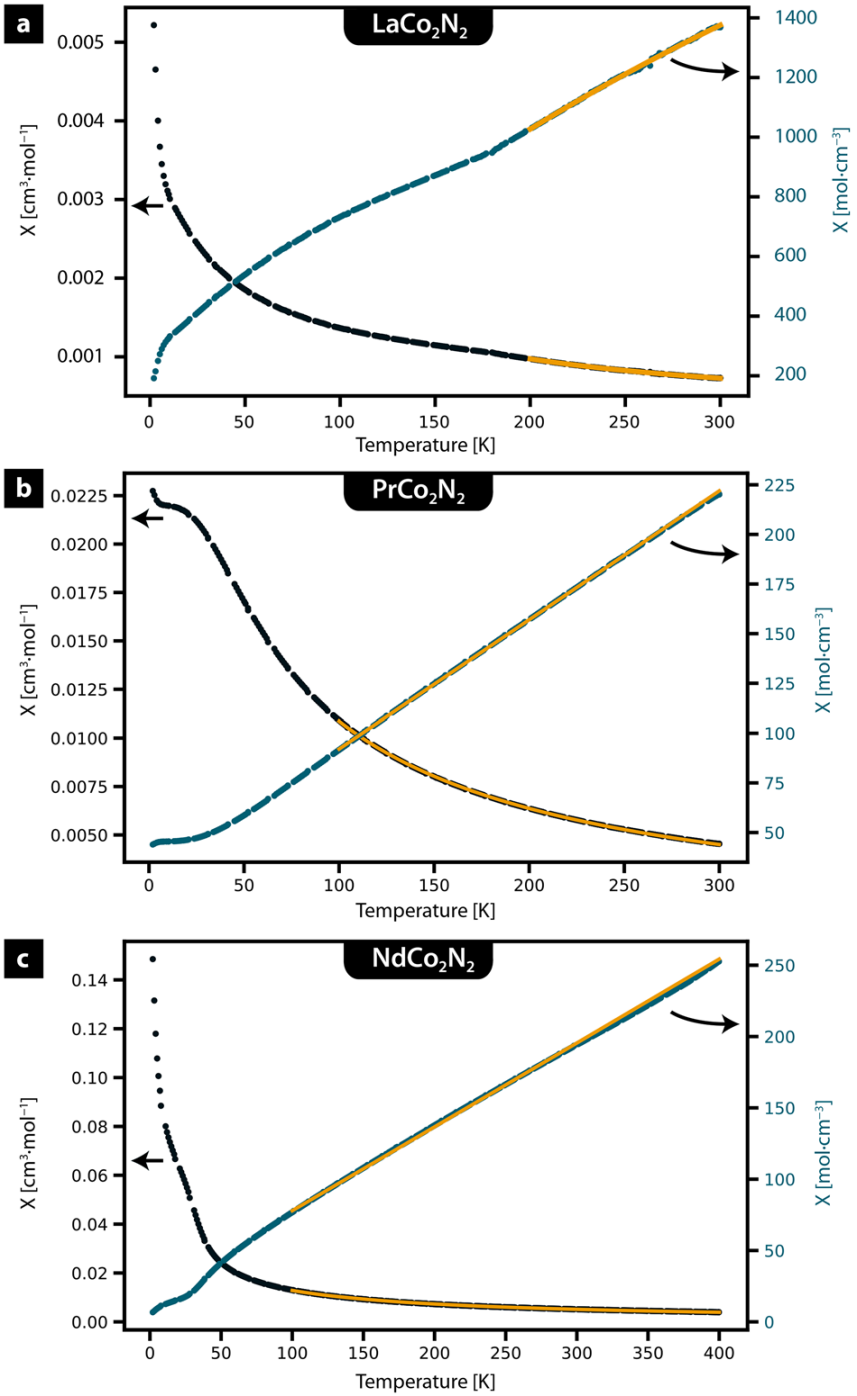
Magnetic susceptibility of *Ln*Co_2_N_2_ materials recorded in fields of 30 kOe with LaCo_2_N_2_ in (a), PrCo_2_N_2_ in (b), and NdCo_2_N_2_ in (c). The molar susceptibility in cgs units is displayed as black circles, the inverse as blue circles, and the Curie‐Weiss fits as orange lines.

At high temperatures, the susceptibility of PrCo_2_N_2_ (Figure 3b) obtained in a field of 30 kOe follows the Curie‐Weiss law, and fitting resulted in a Weiss temperature of *Θ*  =  −41.7(2) K and an effective magnetic moment of *µ_eff_* = 3.509(1) µ_B_. The effective moment closely matches the theoretical value of Pr^3+^‐ions of *µ_eff_* = 3.58 µ_B_ [43], while the negative Weiss temperature indicates antiferromagnetic interactions. The measured *µ_eff_* therefore indicates that Co does not contribute to the magnetism, in line with measurements of the La‐compound. At low temperatures, the susceptibility diverges from simple Curie‐Weiss behavior and plateaus below 25 K. This, however, likely reflects the crystal field splitting of the ^3^H_4_ ground state stemming from the Pr^3+^:4f^2^ electron configuration rather than exchange‐interactions or magnetic ordering. For the non‐Kramers ion, it is well known that the crystal field can lead to a nonmagnetic *M*_J_ = 0 ground state followed by a series of doublets and triplets [44]. The absence of magnetic ordering is further corroborated by powder neutron diffraction data (Figure S3), where no magnetic reflections were detected in difference data between measurements made at 50 and 1.6 K.

#### 2.4.3 NdCo_2_N_2_

The magnetic susceptibility of NdCo_2_N_2_ at high temperatures and fields of 30 kOe shows Curie‐Weiss‐type behavior stemming from Nd^3+^‐ions. A fit in the range from 100 to 400 K resulted in a Weiss temperature of *Θ* = −32.5(3) K and an effective magnetic moment of *µ*_eff_ = 3.688(3) µ_B_, which aligns well with the theoretical value of the Nd^3+^‐ion of *µ*_eff_ = 3.62 µ_B_ [43]. The Co d‐electrons are therefore also itinerant in the NdCo_2_N_2_.

At low temperatures, a small NdN byproduct of ca. 8% (see Figure 3c) leads to a ferromagnetic transition below *T*_c_ = 50 K, as visible in the susceptibility data as well as in the field‐dependent magnetisation (Figure S3), shrouding possible magnetic ordering of the Nd‐compound at lower temperatures [45]. Synthesis optimization of NdCo_2_N_2_ led to a reduction of the NdN phase fraction, but complete reaction was not achieved, which may be due to sluggish reaction kinetics of inert NaCl‐type NdN with cobalt metal. The difference powder neutron diffraction data of 1.6 K minus 50 K diffractograms (Figure S3) show a very weak signal at low angles, which may reflect frustrated low‐dimensional magnetic ordering stemming from Nd‐ions arranged on a trigonal lattice (Figure 1c).

### 2.5 Thermal Behavior of *Ln*Co_2_N_2_

Temperature‐dependent powder X‐ray diffractometry was employed to identify the thermal stability and thermal expansion of the *Ln*Co_2_N_2_ (*Ln* = La, Pr, Nd) materials. The La‐ phase was heated in increments of 20°C up to 1000°C, while the Pr‐ and Nd‐phases were heated up to 750°C/ 740°C, respectively, due to capillary breakage that likely was facilitated by reaction of the already decomposed samples with the capillaries. Diffractograms were recorded at each temperature step at constant temperature. LaCo_2_N_2_ decomposed at a temperature of 680°C into LaN and Co metal, while PrCo_2_N_2_ decomposed at 670°C into PrN and Co metal, and NdCo_2_N_2_ decomposed at 620°C into NdN and Co metal (Figures S4–S6) [42, 46]. The lattice parameter variation of each compound was monitored via fitting of the powder X‐ray patterns with the Rietveld method. The coefficients of thermal expansion (CTE, *α*) for all phases were obtained via fitting a linear regression on the obtained temperature‐dependent lattice parameters *a*, *c*, and *V* and are summarized in Table 2.

**TABLE 2:** Linear expansion coefficient *α* of *Ln*Co_2_N_2_ (*Ln* = La, Pr, Nd) for the lattice parameters *a*, *c*, and the corresponding cell volume *V*.

	LaCo_2_N_2_	PrCo_2_N_2_	NdCo_2_N_2_
*α_a_* · 10^−5^ [K^−1^]	1.36(1)	1.41(2)	1.44(2)
*α_c_* · 10^−6^ [K^−1^]	6.2(1)	6.0(2)	8.6(2)
*α_V_* · 10^−5^ [K^−1^]	3.35(4)	3.45(4)	3.77(5)

The obtained CTEs for *Ln*Co_2_N_2_ (*Ln* = La, Pr, Nd) are similar and show anisotropic thermal behavior; the expansion in the *a* and *b* directions is roughly twice the amount of the expansion in the *c* direction. The materials display a thermal behavior similar to other transition metal nitrides such as Ca_2_NiN_2_ (*α*_a_ = 1.8(1) · 10^−5^ K^−1^, *α*_c_ = 7.1(6) · 10^−6^ K^−1^), LaReN_3_ (*α*_a_ = 13(1) · 10^−6^ K^−1^), and *Ln*_2_ReN_4_ (*Ln* = Pr, Nd) (*α* = 5 · 10^−6^–10 · 10^−6^ K^−1^) [27, 28, 29].

### 2.6 Density Functional Theory

**FIGURE 4:**
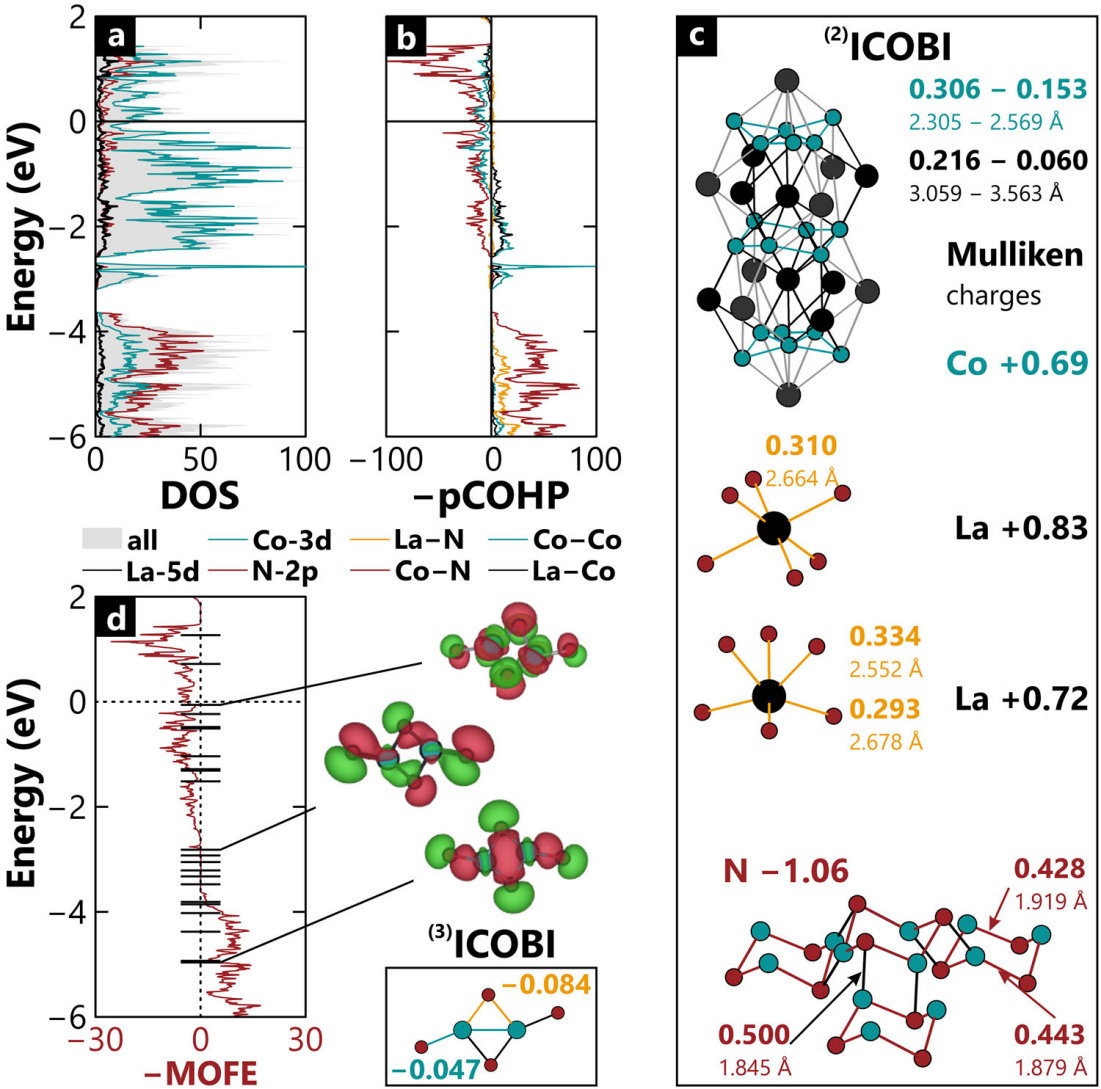
(a) total and orbital‐projected densities of states (DOS), (b) projected orbital Hamilton populations (−pCOHP) and (d) molecular orbital formation energy (−MOFE) diagrams of LaCo_2_N_2_; the Fermi level is located at 0 eV, while two‐center integrated crystal orbital bond indices (^(2)^ICOBI/bond) as well as Mulliken charges have been included in (c).

In the ionic bonding picture of *Ln*Co_2_N_2_ ≡ (*Ln* ^3+^)(Co^+^)(Co^2+^)(N^3−^)_2_, cobalt is in mixed valence states +I/II; however, the results of the structural analysis implied a more complex bonding situation, where the metallic ground state of the compound leads to an intervalent +1.5 state for the cobalt atoms. Quantum‐chemical electronic structure analysis was therefore carried out for LaCo_2_N_2_ (Figure 4).

The density of states curves (DOS) indicate that the Fermi level is at a non‐zero region, suggesting a metallic ground state, in accord with the observed magnetic properties. The DOS in the energy regions around the Fermi level originates largely from the Co‐3d and N‐2p atomic orbitals with minor contributions from the La‐5d states. The Mulliken charges of the Co and La sites indicate polar‐covalent metal–nitrogen interactions, while the two‐center ^(2)^ICOBI/bond values are in a range encountered [47, 48] for ionocovalent interactions (Figure 4c). Notably, a comparison of the Mulliken charges and ^(2)^ICOBI/bond values also reveals that the polar character is more pronounced for the La−N bonds than for the Co−N interactions, in agreement with Pearson's electronegativities of nickel (4.40) and lanthanum (3.1) [49]. Also, the lanthanum positions differ in the respective Mulliken charges, whereas all cobalt sites correspond to the same Mulliken charge. That outcome is in full line with the results of the structural analysis, because different oxidation states of cobalt should also translate into dissimilar site symmetries, which is not the case (Table 1).

In addition to the metal−nitrogen bonds, there are also La−Co and Co−Co interactions, whose ^(2)^ICOBI/bond values point to a metallic bonding character [47]. Accordingly, the structure model of LaCo_2_N_2_ is composed of an intermetallic network that is oxidized by the nitride anions (Figure 4c). This agrees with Mulliken charges of the cobalt atoms (+0.69) being smaller than the formal charges of +1 and +2. Furthermore, the metal−metal bonding interactions somewhat counterbalance the antibonding metal−nitrogen states (Figure 4b), leading to an enhancement of the energetic contribution to net total energy [50, 51]. However, it is still striking that the cobalt and nitrogen atoms establish puckered sheets where every cobalt atom is surrounded by three nitrogen atoms, while, for instance, Co^+^ is solely coordinated by two oxygen ligands within oxides [52, 53].

Analysis of the Fragment Molecular Orbitals (FMOs) and the Molecular Orbital Formation Energy (−MOFE) for a [Co_2_N_4_] unit (Figure 4d) reveals additional influences on the geometry of the puckered cobalt‐ and nitrogen‐containing sheets. An inspection of the three‐center ^(3)^ICOBI values reveals a multicenter character [47, 54] for the Co−Co−N (−0.047) and Co−N−Co (−0.084) moieties. The ^(3)^ICOBI value of the former unit is smaller than that of the latter fragment, because the Co−Co bonds exhibit a more delocalized character, which translates into the smaller ^(3)^ICOBI value. That combination, i.e., the simultaneous presence of a polar, covalent, and delocalized character for the Co−N, is reminiscent of a multicenter nature, which is well recognized for molecular units [55, 56, 57, 58]. Also, an analysis of the −MOFE diagram reveals a clear transition from stabilizing states, which originate from predominantly bonding interactions, to destabilizing ones that arise from mostly antibonding contributions. Such a transition from bonding to antibonding states is also evident from an examination of the −pCOHP diagram and is an attribute of the polar‐covalent bonding character [59]. Furthermore, there are also FMOs, which correspond to −IMOFE values close to zero and may be assigned to the nitrogen lone pairs. These nitrogen lone pairs may therefore be stereochemically active, contributing to the puckered geometry of the cobalt layers.

## 3 Conclusion

The preparation of *Ln*Co_2_N_2_ (*Ln* = La, Pr, Nd) demonstrates that high‐pressure synthesis gives access to nitridometallates with extended anionic networks, greatly proliferating the structural diversity and enabling the stabilization of small *A*:*M* ratios. The synthesis conditions of 8 GPa as well as common starting materials make this approach transferable to other transition metal systems. The combination of single‐crystal X‐ray diffraction as well as neutron diffraction shows that *Ln*Co_2_N_2_ (*Ln* = La, Pr, Nd) are fully nitrided, resulting in (formally) mixed valent Co^+I/+II^ oxidation states. The metallic ground state and the presence of an extended nitridometallate network place the materials on the border of intermetallics and classic nitridometallates.

Our report on *Ln*Co_2_N_2_ (*Ln* = La, Pr, Nd) serves as a starting point for the systematic exploration of nitridometallates with extended anionic networks. A wide variety of chemical compositions is available by similar synthetic approaches; substitution of electropositive cations and transition metals yields a rich space for materials discovery. The relation of electronic ground state, e.g., driving electron localization, with structural features such as coordination polyhedra and network topology gives further incentive for exploration of this exciting class of compounds.

## 4 Experimental Section/Methods

### 4.1 Synthesis of LnCo_2_N_2_

The title compounds were prepared according to Equation 1 under high pressure/ high temperature conditions achieved with a Voggenreiter 1000 t hydraulic press (Mainleus, Germany) and the multianvil technique. For the preparation of LaCo_2_N_2_, PrCo_2_N_2_, and NdCo_2_N_2_ stoichiometric amounts of LaN (32.2 mg, 0.211 mmol), PrN (35.1 mg, 0.227 mmol) or NdN (32.7 mg, 0.207 mmol), two equivalents of Co powder (26.1 mg, 0.443 mmol or 28.1 mg, 0.477 mmol or 25.6 mg, 0.434 mmol, respectively) in 10% excess and one equivalent of NaN_3_ (27.4 mg, 0.421 mmol or 29.5 mg, 0.454 mmol or 26.9 mg, 0.414 mmol, respectively) were mixed under inert conditions of an Ar‐filled glovebox (*c*(O_2_/H_2_O) < 1 ppm) and ground to form a homogeneous powder, which was transferred into a Cu‐foil (0.025 mm thickness, 99.999%, Puratronic, AlfaAesar) lined *h*‐BN crucible and placed into the sample octahedron. The Cr_2_O_3_‐doped MgO sample octahedron (6.5% doping, Ceramic Substrates & Components Ltd., Newport, UK) served as the pressure medium with a sample size of 18/11, representing the octahedron edge length to tungsten carbide cube edge truncation length in mm. Reactions were carried out at 8 GPa and 850°C with a ramp‐up time of 60 min, a dwell time of 120 min for La/Pr and 180 min for Nd, and a ramp‐down time of 30 min. Heating was enabled via two graphite sleeves (Schunk Kohlenstofftechnik GmbH, Geißen, Germany) to diminish thermal gradients. The literature contains further information about the setup [60].

### 4.2 Synthesis of LnN

The *Ln*N (*Ln* = La, Pr, Nd) starting materials were synthesized in a similar manner as described by Ettmayer et al. by reaction of the respective lanthanide metals and N_2_ in a high‐frequency furnace [46]. For the reaction, the metal pieces were filed into fine shavings and placed in a W crucible. The crucible was heated for 14 h under a dried N_2_ atmosphere at a temperature of 1200°C for La and Pr, and 1400°C for Nd. Phase purity was confirmed by PXRD.

### 4.3 Single‐Crystal X‐Ray Diffraction (SCXRD)

The samples were examined for single crystals under a light microscope in anhydrous paraffin oil. Suitable crystals were selected, isolated, and mounted on MicroMounts (10 µm, Mitegen, New York, USA) and then measured using a D8 Venture diffractometer (Bruker, Billerica, MA, USA) equipped with Mo‐*Kα_1_* radiation (*λ* = 0.71073 Å), a CMOS detector, and a graphite monochromator. The collected data were indexed, integrated using the narrow‐frame algorithm, and absorption corrected with the multi‐scan method in the APEX6 software [34]. Space group determination, structure solution by intrinsic phasing, and structure refinement were performed using the XPREP, SHELXT, and SHELXS implementations within APEX6 [61, 62, 63, 64]. All atoms were refined anisotropically, and the final structure was visualized using VESTA [65].

### 4.4 Powder X‐Ray Diffraction (PXRD) and Powder Neutron Diffraction (PND)

In‐house powder X‐ray diffraction patterns were recorded with a StadiP diffractometer (Stoe & Cie, Darmstadt, Germany) in parafocused Debye‐Scherrer geometry equipped with Ag‐K*α*_1_ radiation (*λ* = 0.5594217 Å), a Ge(111) monochromator, and a Si‐strip detector (Mythen1K‐detector, Dectris, Baden, Switzerland). The samples were sealed in glass capillaries (Hilgenberg GmbH, Malsfeld, Germany) with a diameter of 0.3 mm, which served as sample holders. Data were collected with a range of 2 < 2*Θ* < 60° for each sample.

Powder neutron diffraction data were collected using the high‐intensity two‐axis neutron powder diffractometer D20 of the Institut Laue‐Langevin (ILL) in Grenoble, France [66]. The structural details were determined using the high‐resolution option of D20 at a takeoff angle of 90° and a wavelength of *λ * =  1.54 Å delivered by a germanium monochromator. Data were collected within a range of 0 < 2*Θ* < 153° for each sample, and co‐refinements of X‐ray and neutron diffraction data were carried out using TOPAS‐Academic V6 software [67, 68, 69, 70, 71].

To evaluate a possible magnetic ordering, high‐intensity neutron powder diffraction measurements using the 42° takeoff angle and a graphite monochromator were collected at 1.6 and 50 K using a wavelength of 2.41 Å.

### 4.5 Temperature‐Dependent Powder X‐Ray Diffraction (HTXRD)

Temperature‐dependent powder X‐ray diffraction (HTXRD) experiments were performed on a Stoe StadiP diffractometer (Stoe & Cie, Darmstadt, Germany) equipped with a Stoe resistance graphite furnace for precise temperature control. The compounds were finely ground and loaded into a silica capillary (outer diameter: 0.4 mm, Hilgenberg GmbH, Malsfeld, Germany). Samples of LaCo_2_N_2_ and NdCo_2_N_2_ were heated from 60°C to 1000°C in 20°C increments, whereas PrCo_2_N_2_ was heated from 50°C to 950°C in 20°C increments under an Ar atmosphere. At each temperature step, a diffraction pattern was recorded over a 2*Θ* range of 3°–77° using Ag‐K*α*_1_ radiation (*λ* = 0.5594217 Å) filtered with a Ge(111) monochromator. An image plate position‐sensitive detector (IP‐PSD) was used to capture the diffraction intensities. TOPAS‐Academic V6 was used to analyze changes in the lattice parameters as a function of temperature with the Rietveld method.

### 4.6 Scanning Electron Microscopy (SEM‐EDX)

The sample was investigated using a scanning electron microscope (SEM, Dualbeam Helios Nanolab G3 UC, Fei, Hillsboro, Oregon, USA) at an accelerating voltage of 18 kV with an energy‐dispersive X‐ray detector (model X‐Max 80 SDD, Oxford Instruments, Abingdon, UK). Spatially resolved elemental analysis (EDX, energy‐dispersive X‐ray spectroscopy) was performed and processed with AZtec software [72].

### 4.7 Magnetic Measurements

For magnetic measurements, the samples were loaded into polypropylene capsules with known diamagnetic contribution (Quantum Design, San Diego, CA, USA), and the measurements were conducted on a Physical Properties Measurement System (PPMS‐9, Quantum Design, San Diego, CA, USA) using a vibrating sample magnetometer (VSM) option. The isothermal magnetization was measured at 2 and 300 K with external fields between H = ±50 kOe. The susceptibility was determined at 100 and 30 000 Oe while the temperature varied between 2 and 300 K for LaCo_2_N_2_ and PrCo_2_N_2_, and between 2 and 400 K for NdCo_2_N_2_.

### 4.8 Computational Details

To provide an insight into the electronic structure of the herein reported nitrides, quantum‐chemical investigations have been accomplished for the example of LaCo_2_N_2_ in a non‐spin‐polarized regime. A non‐spin‐polarized approach was appropriate here, despite that the respective spin state can influence the size, the nature of the electronic interactions and the potential of a transition metal to some extent [73]; the experimentally determined structure model, however, has been well reproduced in the framework of the full structural optimizations that included lattice parameters as well as atomic positions and were accomplished prior to the electronic structure investigations. This outcome means that the spin states of the diverse atoms have been adequately described in the computations so that the electronic structure analysis was carried out. All electronic structure computations were completed by means of the projector augmented wave method [74] within the Vienna ab initio simulation package [75, 76, 77, 78, 79] (VASP) code. In all computations, correlation and exchange were described by the generalized gradient approximation [80] (GGA−PBE), whereas a 7 × 7 × 3 **k**‐points set was employed to sample the Brillouin zone for reciprocal space integrations. The energy cut of the plane waves was 500 eV, and all computations were considered to be converged as the energy difference between two iterative steps fell below 10^−8^ eV (and 10^−6^ eV) of the electronic (and ionic) relaxation. In order to gain an access to the bonding scenario in LaCo_2_N_2_, different kinds of bonding indicators were used, i.e. the Mulliken [81] charges, the projected crystal orbital Hamilton populations [82, 83] (−pCOHP), the crystal orbital bond indices [84] (COBI) and the fragment molecular orbitals [85] of a specific Co_2_N_4_ unit (FMOs). The bonding indicators were extracted from the results of the plane wave‐based computations with the aid of the Local Orbital Basis Suite toward Electronic‐Structure Reconstruction [56, 86, 87] (LOBSTER) program. Furthermore, the densities of states (DOS) and −pCOHP diagrams were analyzed and plotted by using the *wxdragon* [88] code, while the FMOs were visualized by means of the aforementioned program VESTA [65].

## Author Contributions

**Jonas M. Albrecht**: investigation, validation. **Clemens Ritter**: investigation, validation. **Dominik Werhahn**: investigation, validation. **Simon Steinberg**: investigation, visualization, formal analysis, writing – original draft, validation. **Nina A. M. Prinz**: conceptualization, investigation, formal analysis, data curation, visualization, writing – original draft, validation. **Simon D. Kloß**: validation, conceptualization, investigation, funding acquisition, writing – original draft, writing – review and editing, visualization, project administration, resources, supervision, data curation, formal analysis, methodology.

## Conflicts of Interest

The authors declare no conflicts of interest.

## Supporting information




**Supporting File**: advs76531‐sup‐0001‐SuppMat1.pdf.

## Data Availability

All raw data will be made available via the Open Data LMU repository under a Creative Commons license. The crystallographic data for the structures reported in this work have been deposited with the Inorganic Crystal Structure Database (ICSD) at FIZ Karlsruhe – Leibniz Institute for Information Infrastructure. The data can be obtained free of charge via the joint access service of FIZ Karlsruhe and the Cambridge Crystallographic Data Centre (CCDC) at https://www.ccdc.cam.ac.uk/structures/ (or https://icsd.fiz‐karlsruhe.de), quoting the deposition numbers CSD‐2548415, CSD‐2548416, CSD‐2548417 for models based on SCXRD data and CSD‐2548412, CSD‐2548412, and CSD‐2548412 for co‐refined models from PXRD and PND data.
