# Supplementary material for: Extended Anionic Network in Mixed‐Valent Nitridocobaltates(I/II) LnCo2N2 (Ln = La, Pr, Nd)
Source: Adv Sci (Weinh). 2026 Jul 9:e76531. Online ahead of print. doi: 10.1002/advs.76531 (PMC13348335; doi:10.1002/advs.76531)
Supplement: Supplementary file 1 — Supporting File: advs76531‐sup‐0001‐SuppMat1.pdf. [file ADVS-9999-e76531-s001.pdf]

Supporting Information

Extended Anionic Network in Mixed-Valent Nitridocobaltates(I/II) *Ln*Co_2N_2 (*Ln* = La, Pr, Nd)

*Nina A. M. Prinz,[a] Jonas M. Albrecht,[a] Dominik Werhahn,[a] Simon Steinberg,[b] Clemens Ritter,[c] and Simon D. Kloß [a]**

a Department Chemistry, LMU Munich, Munich, Germany
b Institute of Inorganic Chemistry, RWTH Aachen University, Aachen, Germany
c Institut Laue-Langevin, Grenoble, France

Table of Contents

1 SEM-EDX 2
2 Single-crystal X-ray diffraction 3
2.1 Crystallographic Tables 3
2.2 Tables for $LaCo_2N_2$ 4
2.3 Tables for $PrCo_2N_2$ 5
2.4 Tables for $NdCo_2N_2$ 6
3 Bond valence sum calculations (BVS) 7
4 Co-refinement of powder x-ray and neutron diffraction data 8
4.1 Co-refinement of PXRD and PND 8
4.2 Tables for $LaCo_2N_2$ 9
4.3 Tables for $PrCo_2N_2$ 10
4.4 Tables for $NdCo_2N_2$ 12
5 Magnetometry 14
6 Temperature-dependent powder X-ray diffraction 15
7 References 18

1 SEM-EDX

The morphology and composition of *Ln*Co_2N_2 with *Ln* = La, Pr, Nd were analyzed using SEM-EDX. Several crystals of the bulk samples were characterized for each phase. For this analysis, all phases were washed with 0.1 M HCl to remove the Na resulting from the synthesis with NaN_3. However, due to sensitivity to oxygen and hydrolysis, the washed samples were only used for this analysis. Oxygen and chlorine contents were disregarded to determine the compositions of *Ln*, Co, and N.

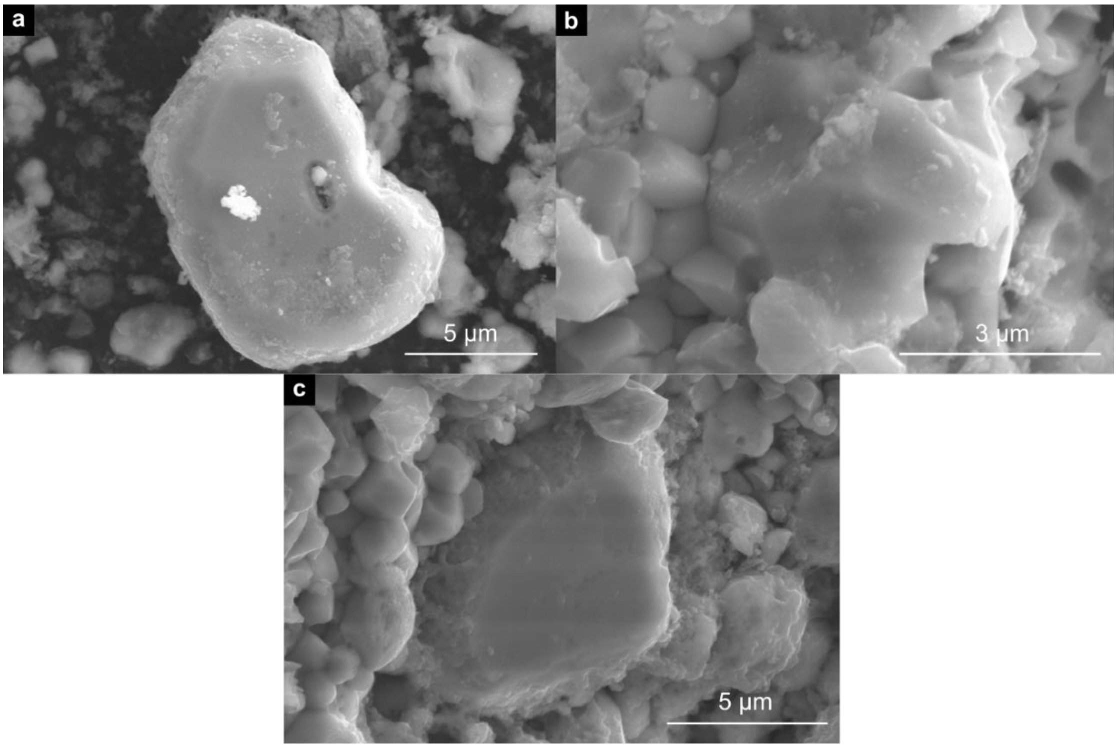

Figure S1: SEM micrograph of a) $LaCo_2N_2$, b) $PrCo_2N_2$, and c) $NdCo_2N_2$ crystallites.

Table S1: Results of the EDX measurements given in atom-% on different crystals from each bulk sample of $LaCo_2N_2$, $PrCo_2N_2$, and $NdCo_2N_2$.

Measurement	La	Co	N	Pr	Co	N	Nd	Co	N
1	19.3	40.0	40.7	20.5	41.5	37.9	19.7	39.2	41.1
2	21.0	41.9	37.0	19.1	38.0	43.0	19.7	39.0	41.3
3	18.2	37.3	44.5	19.0	38.2	42.8	22.9	44.2	32.9
4	21.4	41.7	36.9	20.6	42.4	37.1	16.9	34.4	48.7
5	21.0	43.0	36.0	20.0	40.7	39.4	17.7	34.8	47.5
6	22.0	47.4	30.5	13.6	40.0	46.4	18.8	37.1	44.1
7	27.1	55.9	17.1	25.3	49.2	25.4	23.8	45.5	30.7
8	21.1	50.1	28.8	17.9	36.5	45.6			
9				15.1	46.5	38.4			
10				18.6	39.3	42.1			
**ø**	**21.4**	**44.6**	**34.0**	**19.0**	**41.2**	**39.8**	**19.9**	**39.2**	**40.9**
Theory	20.0	40.0	40.0	20.0	40.0	40.0	20.0	40.0	40.0

2 Single-crystal X-ray diffraction

2.1 Crystallographic Tables

Table S2: Crystallographic data of $LaCo_2N_2$, $PrCo_2N_2$ and $NdCo_2N_2$ from SCXRD refinement. Standard deviations are given in brackets.

**Crystal data**			
Formula	$LaCo_2N_2$	$PrCo_2N_2$	$NdCo_2N_2$
Formula mass [g · mol^{-1}]	284.79	286.79	290.12
Crystal system		trigonal	
Space group		$R\bar{3}$ (no. 148)	
Cell parameters [Å]	a, b = 6.5664(6)	a, b = 6.4955(5)	a, b = 6.4702(3)
	c = 15.568(2)	c = 15.3677(16)	c = 15.3123(16)
Cell volume [Å^3]	581.31(13)	561.52(10)	555.14(8)
Z		9	
F(000)	1125	1143	1152
Calc. density [g · cm^{-3}]	7.322	7.633	7.81
Absorption coefficient μ [mm^{-1}]	28.579	31.991	33.655
**Data collection**			
Diffractometer		Bruker D8 Venture	
Radiation [Å]		Mo-K_α; Microfocus X-ray Source	
Temperature [K]		299(2)	
θ-range [Deg.]	3.815, 28.251	3.857, 26.352	3.872, 30.409
hkl-range	$-8 \le h \le 8$<br>$-8 \le k \le 8$<br>$-20 \le l \le 20$	−8 ≤ h ≤ 8<br>−7 ≤ k ≤ 8<br>−19 ≤ l ≤ 19	$-9 \le h \le 9$<br>$-9 \le k \le 9$<br>$-21 \le l \le 21$
Measured reflections	2761	2492	4024
Unique reflections	323	253	374
Absorption correction		Multi-Scan, SADABS	
Range of transmission	0.6557, 0.7477	0.6137, 0.7473	0.6568, 0.7481
R_{int}, R_σ	0.0316, 0.0165	0.047, 0.0234	0.0316, 0.0152
**Refinement**			
Observed reflections	304	225	350
Reflections criterion	$I > 2\sigma(I)$	$I > 2\sigma(I)$	$I > 2\sigma(I)$
No. refined parameters	25	25	24
No. Restraints	0	0	0
Refinement method		Full-matrix least-squares	
GoF	1.334	1.196	1.222
Final R / $I > 2\sigma(I)$	R_1 = 0.016, wR_2 = 0.032	R_1 = 0.021, wR_2 = 0.030	R_1 = 0.015, wR_2 = 0.028
Final R (all data)	R_1 = 0.018, wR_2 = 0.033	R_1 = 0.027, wR_2 = 0.031	R_1 = 0.017, wR_2 = 0.029
Largest diff. peak and hole [e·Å^{-3}]	1.044, −0.772	0.998, −1.287	1.286, −1.364

[a] Weighting scheme for $LaCo_2N_2$: calc. $w = \frac{1}{s^2(Fo^2)+(0.0090P)^2+3.6474P}$ where $P = \frac{(Fo^2+2Fc^2)}{3}$

[b] Weighting scheme for $PrCo_2N_2$: calc. $w = \frac{1}{s^2(Fo^2)+(0.0015P)^2+7.9179P}$ where $P = \frac{(Fo^2+2Fc^2)}{3}$

[c] Weighting scheme for $NdCo_2N_2$: calc. $w = \frac{1}{s^2(Fo^2)+(0.0082P)^2+3.7732P}$ where $P = \frac{(Fo^2+2Fc^2)}{3}$

2.2 Tables for $LaCo_2N_2$

Table S3: Anisotropic atomic displacement parameters for $LaCo_2N_2$. Standard deviations are given in brackets.

Atom	U_{11} [Å^2]	U_{22} [Å^2]	U_{33} [Å^2]	U_{12} [Å^2]	U_{13} [Å^2]	U_{23} [Å^2]
La1	0.0050(2)	0.0050(2)	0.0054(3)	0.00252(10)	0	0
La2	0.00459(17)	0.00459(17)	0.0042(2)	0.00230(8)	0	0
Co1	0.0039(3)	0.0050(3)	0.0050(3)	0.0024(2)	-0.0009(2)	0.0005(2)
N1	0.0051(17)	0.0058(17)	0.0066(17)	0.0019(15)	-0.0002(14)	-0.0004(14)

Table S4: Interatomic distances and bonding angles in the structure of $LaCo_2N_2$. Symmetry-related identical distances have been omitted. Standard deviations are given in brackets.

Atoms	Distance [Å]	Atoms	Angle [Deg.]
La1–N1	2.648(3)	N1–La1–N1	180.0
La1–Co1	3.2445(7)	N1–La1–N1	97.64(10)
La2–N1	2.551(4)	N1–La1–N1	82.36(10)
La2–N1	2.667(4)	N1–La2–N1	151.68(8)
La2–Co1	3.0729(7)	N1–La2–N1	111.51(7)
La2–Co1	3.1884(8)	N1–La2–N1	83.82(16)
Co1–N1	1.859(4)	N1–La2–N1	82.36(12)
Co1–N1	1.899(4)	N1–La2–N1	74.52(13)
Co1–N1	1.964(4)	N1–Co1–N1	148.52(18)
Co1–Co1	2.3341(11)	N1–Co1–N1	106.1(2)
Co1–Co1	2.5628(7)	N1–Co1–N1	104.81(14)
Co1–Co1	2.5763(11)		

2.3 Tables for $PrCo_2N_2$

Table S5: Anisotropic atomic displacement parameters for $PrCo_2N_2$. Standard deviations are given in brackets.

Atom	U_{11} [Å^2]	U_{22} [Å^2]	U_{33} [Å^2]	U_{12} [Å^2]	U_{13} [Å^2]	U_{23} [Å^2]
Pr1	0.0052(3)	0.0052(3)	0.0055(5)	0.00258(16)	0	0
Pr2	0.0042(2)	0.0042(2)	0.0047(4)	0.00208(12)	0	0
Co1	0.0044(5)	0.0060(5)	0.0047(5)	0.0029(4)	-0.0011(4)	0.0004(3)
N1	0.007(3)	0.006(3)	0.005(3)	0.004(2)	0.001(2)	0.001(2)

Table S6: Interatomic distances and bonding angles in the structure of $PrCo_2N_2$. Symmetry-related identical distances have been omitted. Standard deviations are given in brackets.

Atoms	Distance [Å]	Atoms	Angle [Deg.]
Pr1–N1	2.594(5)	N1–Pr1–N1	180.0
Pr1–Co1	3.2129(9)	N1–Pr1–N1	98.22(15)
Pr2–N1	2.498(5)	N1–Pr1–N1	81.78(15)
Pr2–N1	2.635(5)	N1–Pr2–N1	152.29(11)
Pr2–Co1	3.0336(10)	N1–Pr2–N1	111.79(10)
Pr2–Co1	3.1354(10)	N1–Pr2–N1	82.8(2)
Co1–N1	1.858(5)	N1–Pr2–N1	81.91(18)
Co1–N1	1.896(5)	N1–Pr2–N1	75.91(19)
Co1–N1	1.963(5)	N1–Co1–N1	148.8(3)
Co1–Co1	2.3265(17)	N1–Co1–N1	105.5(3)
Co1–Co1	2.5256(10)	N1–Co1–N1	105.0(2)
Co1–Co1	2.5638(15)		

2.4 Tables for $NdCo_2N_2$

Table S7: Anisotropic atomic displacement parameters for $NdCo_2N_2$. Standard deviations are given in brackets.

Atom	U_{11} [Å^2]	U_{22} [Å^2]	U_{33} [Å^2]	U_{12} [Å^2]	U_{13} [Å^2]	U_{23} [Å^2]
Nd1	0.00467(13)	0.00467(13)	0.0052(2)	0.00234(6)	0	0
Nd2	0.00473(10)	0.00473(10)	0.00436(15)	0.00237(5)	0	0
Co1	0.0037(2)	0.0045(2)	0.0045(2)	0.00237(17)	-0.00062(16)	0.00040(16)
N1	0.0037(12)	0.0061(12)	0.0041(13)	0.0019(11)	0.0003(10)	-0.0002(11)

Table S8: Interatomic distances and bonding angles in the structure of $NdCo_2N_2$. Symmetry-related identical distances have been omitted. Standard deviations are given in brackets.

Atoms	Distance [Å]	Atoms	Angle [Deg.]
Nd1–N1	2.571(3)	N1–Nd1–N1	180.0
Nd1–Co1	3.2025(5)	N1–Nd1–N1	98.45(8)
Nd2–N1	2.621(3)	N1–Nd1–N1	81.55(8)
Nd2–Co1	3.0206(5)	N1–Nd2–N1	152.47(6)
Nd2–Co1	3.1215(6)	N1–Nd2–N1	111.96(6)
Co1–N1	1.859(3)	N1–Nd2–N1	82.29(12)
Co1–N1	1.898(3)	N1–Nd2–N1	81.70(9)
Co1–N1	1.968(3)	N1–Nd2–N1	81.96(6)
Co1–Co1	2.3245(9)	N1–Nd2–N1	76.49(10)
Co1–Co1	2.5124(5)	N1–Co1–N1	148.79(14)
Co1–Co1	2.5572(8)	N1–Co1–N1	105.25(17)

3 Bond valence sum calculations (BVS)

Bond valence sum (BVS) calculations (Table S12) were performed for La, Pr, Nd and Co in all compounds using the bond valence parameters R_0 = 2.34 Å for La^{+III}, R_0 = 2.30 Å for Pr^{+III} and Nd^{+III}, and R_0 = 1.84 Å for Co suggested by Brese and O'Keeffe.[1]

The bond valence sum for the *Ln*2 site ($LaCo_2N_2$: 2.94, $PrCo_2N_2$: 2.97 and $NdCo_2N_2$: 3.09) supports the expected oxidation state of Ln^{+III}, while the *Ln*1 site values ($LaCo_2N_2$: 2.58, $PrCo_2N_2$: 2.70 and $NdCo_2N_2$: 2.88) are slightly lower than expected, which gives further evidence for extended metal–metal bonding in the $LnCo_2N_2$ materials. The BVS values ($LaCo_2N_2$: 2.52, $PrCo_2N_2$: 2.53 and $NdCo_2N_2$: 2.51) for Co are expectedly larger than for a mixed valent +I/II state, which is most likely owing to the low coordination number of CN = 3, multiple metal–N bonding, as well as metal–metal bonding, resulting in a bonding situation that is not reflected in the structural models of nitrogen-bound cobalt on which the R_0 value was trained.[1]

Table S9: BVS calculations for $LnCo_2N_2$ (*Ln* = La, Pr, Nd). Bond valence v_i for each interatomic distance as well as the sums are shown. The parameter R_0 = 2.34 Å for La^{+III}, R_0 = 2.30 Å for Pr^{+III} and Nd^{+III}, and R_0 = 1.84 Å for Co were used to calculate v_i.

	**$LaCo_2N_2$**		**$PrCo_2N_2$**		**$NdCo_2N_2$**	
	**Distance [Å]**	**v_i**	**Distance [Å]**	**v_i**	**Distance [Å]**	**v_i**
*Ln*1-N1	2.65	0.43	2.594	0.45	2.571	0.48
	2.65	0.43	2.594	0.45	2.571	0.48
	2.65	0.43	2.594	0.45	2.571	0.48
	2.65	0.43	2.594	0.45	2.571	0.48
	2.65	0.43	2.594	0.45	2.571	0.48
	2.65	0.43	2.594	0.45	2.571	0.48
		**∑ = 2.58**		**∑ = 2.70**		**∑ = 2.88**
*Ln*2-N1	2.551	0.57	2.498	0.59	2.482	0.61
	2.551	0.57	2.498	0.59	2.482	0.61
	2.551	0.57	2.498	0.59	2.482	0.61
	2.667	0.41	2.635	0.40	2.621	0.42
	2.667	0.41	2.635	0.40	2.621	0.42
	2.667	0.41	2.635	0.40	2.621	0.42
		**∑ = 2.94**		**∑ = 2.97**		**∑ = 3.09**
Co1-N1	1.859	0.95	1.858	0.95	1.859	0.95
	1.899	0.85	1.896	0.86	1.898	0.85
	1.964	0.72	1.963	0.72	1.968	0.71
		**∑ = 2.52**		**∑ = 2.53**		**∑ = 2.51**

4 Co-refinement of powder x-ray and neutron diffraction data

4.1 Co-refinement of PXRD and PND

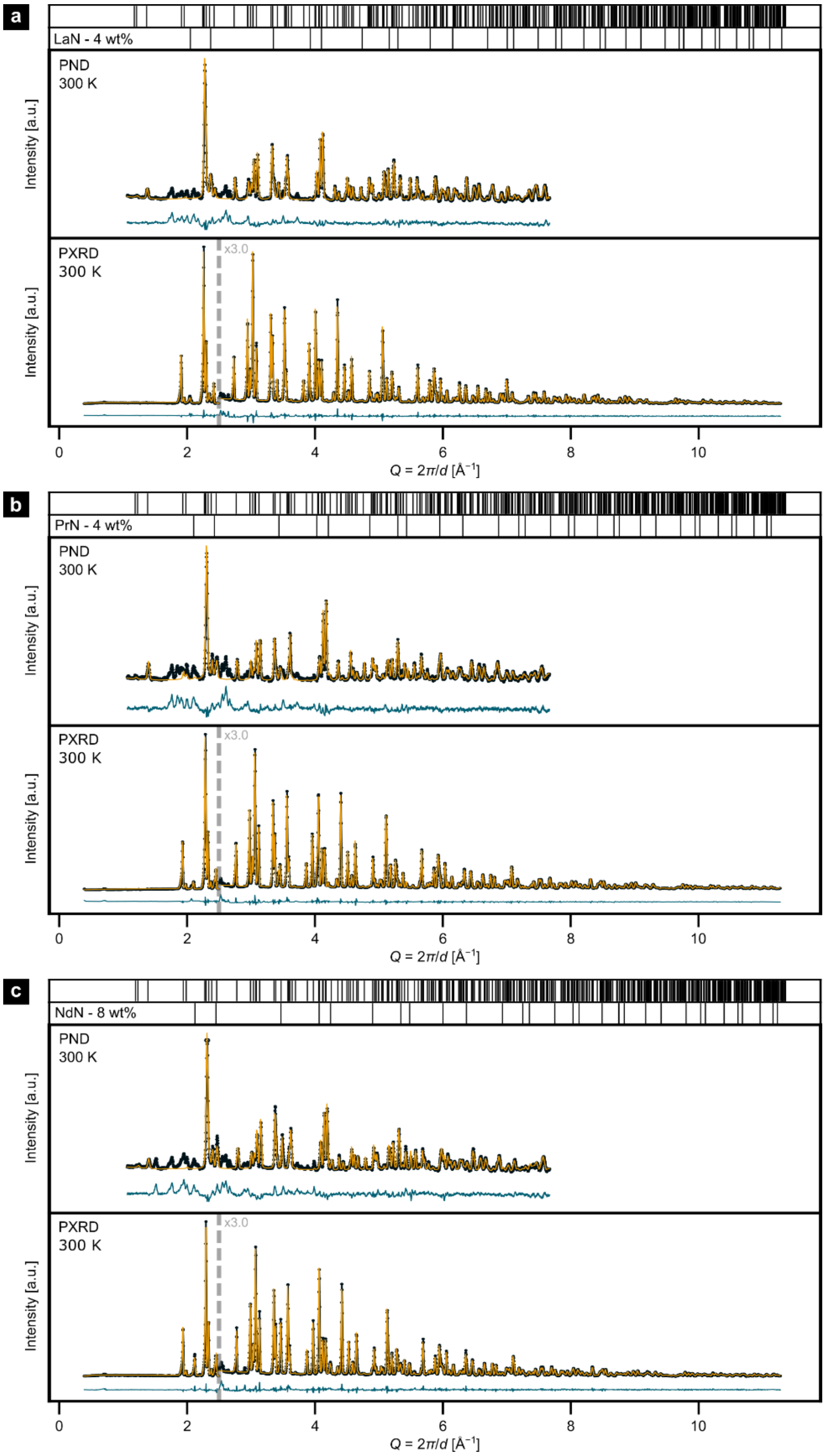

Figure S2: In-house X-ray and neutron co-refinement of a) $LaCo_2N_2$, b) $PrCo_2N_2$ and c) $NdCo_2N_2$ with powder data obtained at 300 K. Black lines indicate the observed data, orange line the Rietveld fit, and the blue line the difference plot. Theoretical reflection positions of *Ln*Co_2N_2 and *Ln*N are marked above in black. Right side of gray dashed line is a 3x magnification region making weak reflections better visible.

4.2 Tables for $LaCo_2N_2$

Table S10: Crystallographic information for $LaCo_2N_2$ from co-refinement of PND and PXRD data at 300 K.

Crystal data		
Formula	$LaCo_2N_2$	
Formula mass [g · mol^{-1}]	284.79	
Crystal system	trigonal	
Space group	$R\bar{3}$ (no. 148)	
Cell parameters [Å]	*a*, *b* = 6.57185(8)	
	c = 15.5650(3)	
Cell volume [Å^3]	582.176(17)	
Z	9	
F(000)	1125	
Calc. density [g · cm^{-3}]	7.311	
**Data collection**		
Diffractometer	Stoe Stadi P	D20 high-intensity two-axis neutron diffractometer
Radiation	Ag Kα_1 (λ = 0.55936 Å)	neutron (λ = 1.54 Å)
Temperature [K]	300	
Q-range	0.39 – 11.28	1.07 – 7.67
Number of Reflections	809	247
**Overall Refinement**		
No. Parameters	12	
No. Constraints	0	
No. Restrains	0	
GOF	3.25	
R_p, R_{wp}, R_{exp}	3.85, 4.65, 1.43	
R_{Bragg}	1.42	

Table S11: Atomic coordinates and equivalent isotropic atomic displacement parameters for $LaCo_2N_2$. Standard deviations are given in brackets.

Atom	*x*	*y*	*z*	Occ	U_{iso} [Å^2]	WYCKOFF	Symm
La1	0	0	0	1	0.0058(5)	3*a*	-3.
La2	0	0	0.36660(8)	1	0.0038(4)	6*c*	3.
Co1	0.0298(4)	0.2401(3)	0.18524(13)	1	0.0042(4)	18*f*	1
N1	0.3836(3)	0.0768(3)	0.08299(15)	1	0.0074(4)	18*f*	1

Table S12: Interatomic distances and bonding angles in the structure of $LaCo_2N_2$. Symmetry-related identical distances have been omitted. Standard deviations are given in brackets.

Atoms	Distance [Å]	Atoms	Angle [Deg.]
La1–N1	2.647(2)	N1–La1–N1	180.0
La2–N1	2.538(2)	N1–La1–N1	98.21(7)
La2–N1	2.662(3)	N1–La1–N1	81.79(7)
La2–Co1	3.073(3)	N1–La2–N1	151.40(8)
Co1–N1	1.877(3)	N1–La2–N1	111.92(7)
Co1–N1	1.905(3)	N1–La2–N1	83.59(7)
Co1–N1	1.974(3)	N1–La2–N1	81.73(6)
		N1–La2–N1	74.78(8)
		N1–Co1–N1	148.80(17)
		N1–Co1–N1	105.49(16)
		N1–Co1–N1	105.21(13)

4.3 Tables for $PrCo_2N_2$

Table S13: Crystallographic information for $PrCo_2N_2$ from co-refinement of PND and PXRD data at 300 K.

**Crystal data**		
Formula	$PrCo_2N_2$	
Formula mass [g · mol^{-1}]	286.79	
Crystal system	trigonal	
Space group	$R\bar{3}$ (no. 148)	
Cell parameters [Å]	a, b = 6.49992(6)	
	c = 15.3644(2)	
Cell volume [Å^3]	562.162(13)	
Z	9	
F(000)	1143	
Calc. density [g · cm^{-3}]	7.624	
**Data collection**		
Diffractometer	Stoe Stadi P	D20 high-intensity two-axis neutron diffractometer
Radiation	Ag Kα_1 (λ = 0.55936 Å)	neutron (λ = 1.54 Å)
Temperature [K]	300	
Q-range	0.39 – 11.28	1.07 – 7.67
Number of Reflections	784	243
**Overall Refinement**		
No. Parameters	12	
No. Constraints	0	
No. Restrains	0	
GOF	4.09	
R_p, R_{wp}, R_{exp}	4.18, 6.75, 1.65	
R_{Bragg}	1.87	

Table S14: Atomic coordinates and equivalent isotropic atomic displacement parameters for $PrCo_2N_2$. Standard deviations are given in brackets.

**Atom**	***x***	***y***	***z***	**Occ**	**U_{iso} [Å^2]**	**WYCKOFF**	**Symm**
Pr1	0	0	0	1	0.0039(4)	3*a*	-3.
Pr2	0	0	0.36547(6)	1	0.0043(3)	6*c*	3.
Co1	0.0317(3)	0.2422(2)	0.18542(10)	1	0.0039(4)	18*f*	1
N1	0.3816(5)	0.0762(4)	0.0820(2)	1	0.0067(5)	18*f*	1

Table S15: Interatomic distances and bonding angles in the structure of $PrCo_2N_2$. Symmetry-related identical distances have been omitted. Standard deviations are given in brackets.

**Atoms**	**Distance [Å]**	**Atoms**	**Angle [Deg.]**
Pr1–N1	2.599(3)	N1–Pr1–N1	180.0
Pr2–N1	2.491(3)	N1–Pr1–N1	98.49(9)
Pr2–N1	2.634(4)	N1–Pr1–N1	81.51(9)
Pr2–Co1	3.035(2)	N1–Pr2–N1	152.14(10)
Co1–N1	1.864(4)	N1–Pr2–N1	111.91(10)
Co1–N1	1.894(4)	N1–Pr2–N1	82.90(10)
Co1–N1	1.968(4)	N1–Pr2–N1	81.60(9)
		N1–Pr2–N1	75.90(12)
		N1–Co1–N1	149.07(17)
		N1–Co1–N1	105.28(16)
		N1–Co1–N1	104.99(14)

4.4 Tables for $NdCo_2N_2$

Table S16: Crystallographic information for $NdCo_2N_2$ from co-refinement of PND and PXRD data at 300 K.

Crystal data		
Formula	$NdCo_2N_2$	
Formula mass [g · mol^{-1}]	290.12	
Crystal system	trigonal	
Space group	$R\bar{3}$ (no. 148)	
Cell parameters [Å]	*a*, *b* = 6.47769(17)	
	c = 15.3081(4)	
Cell volume [Å^3]	556.28(4)	
Z	9	
F(000)	1152	
Calc. density [g · cm^{-3}]	7.794	
**Data collection**		
Diffractometer	Stoe Stadi P	D20 high-intensity two-axis neutron diffractometer
Radiation	Ag Kα_1 (λ = 0.55936 Å)	neutron (λ = 1.54 Å)
Temperature [K]	300	
Q-range	0.39 – 11.28	1.07 – 7.67
Number of Reflections	771	241
**Overall Refinement**		
No. Parameters	12	
No. Constraints	0	
No. Restrains	0	
GOF	3.24	
R_p, R_{wp}, R_{exp}	4.74, 6.15, 1.90	
R_{Bragg}	2.37	

Table S17: Atomic coordinates and equivalent isotropic atomic displacement parameters for $NdCo_2N_2$. Standard deviations are given in brackets.

Atom	*x*	*y*	*z*	Occ	U_{iso} [Å^2]	WYCKOFF	Symm
Nd1	0	0	0	1	0.0048(6)	3*a*	-3.
Nd2	0	0	0.36553(11)	1	0.0037(5)	6*c*	3.
Co1	0.0324(5)	0.2432(5)	0.18545(19)	1	0.0057(6)	18*f*	1
N1	0.3811(4)	0.0736(4)	0.0812(2)	1	0.0093(5)	18*f*	1

Table S18: Interatomic distances and bonding angles in the structure of $NdCo_2N_2$. Symmetry-related identical distances have been omitted. Standard deviations are given in brackets.

Atoms	Distance [Å]	Atoms	Angle [Deg.]
Nd1–N1	2.586(3)	N1–Nd1–N1	180.0
Nd2–N1	2.481(3)	N1–Nd1–N1	98.83(10)
Nd2–N1	2.606(2)	N1–Nd1–N1	81.17(10)
Nd2–Co1	3.019(4)	N1–Nd2–N1	152.28(11)
Co1–N1	1.865(4)	N1–Nd2–N1	112.09(10)
Co1–N1	1.889(5)	N1–Nd2–N1	82.81(10)
Co1–N1	1.985(5)	N1–Nd2–N1	81.04(9)
		N1–Nd2–N1	76.42(12)
		N1–Co1–N1	148.9(2)
		N1–Co1–N1	105.8(2)
		N1–Co1–N1	104.79(18)

5 Magnetometry

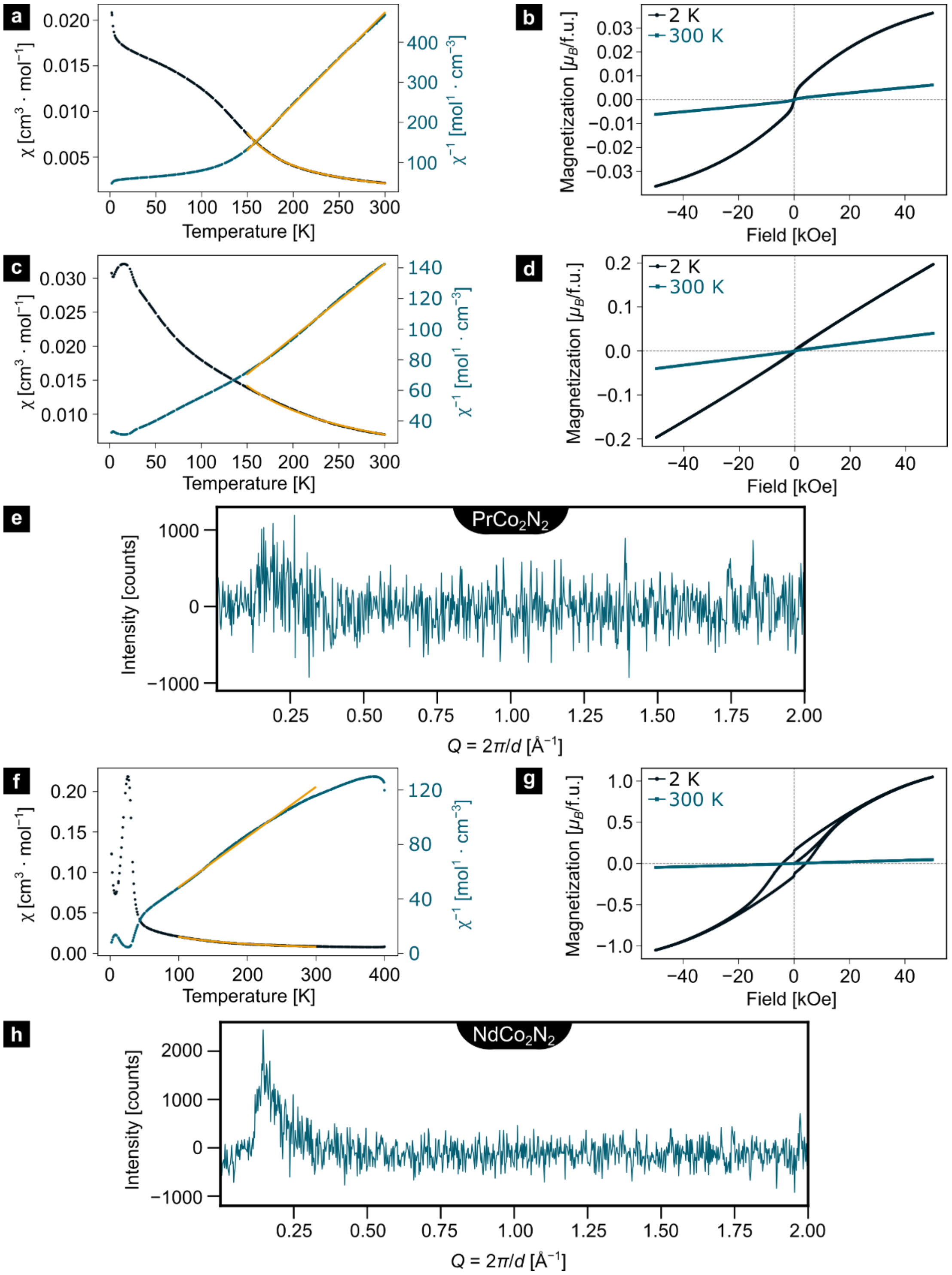

Figure S3: a) Susceptibility measurements of $LaCo_2N_2$ with a Curie-Weiss fit shown in orange at 0.1 T, b) field-dependent measurements of $LaCo_2N_2$ at 300 K and 2 K, c) Susceptibility measurements of $PrCo_2N_2$ with a Curie-Weiss fit shown in orange at 0.1 T, d) field-dependent measurements of $PrCo_2N_2$ at 300 K and 2 K, e) Difference plot of the high-flux measurements at temperatures of 1.6 K and 50 K of $PrCo_2N_2$ (λ = 2.41 Å), f) Susceptibility measurements of $NdCo_2N_2$ with a Curie-Weiss fit shown in orange at 0.1 T, g) field-dependent measurements of $NdCo_2N_2$ at 300 K and 2 K, h) Difference plot of the high-flux measurements at temperatures of 1.6 K and 50 K of $NdCo_2N_2$ (λ = 2.41 Å).

6 Temperature-dependent powder X-ray diffraction

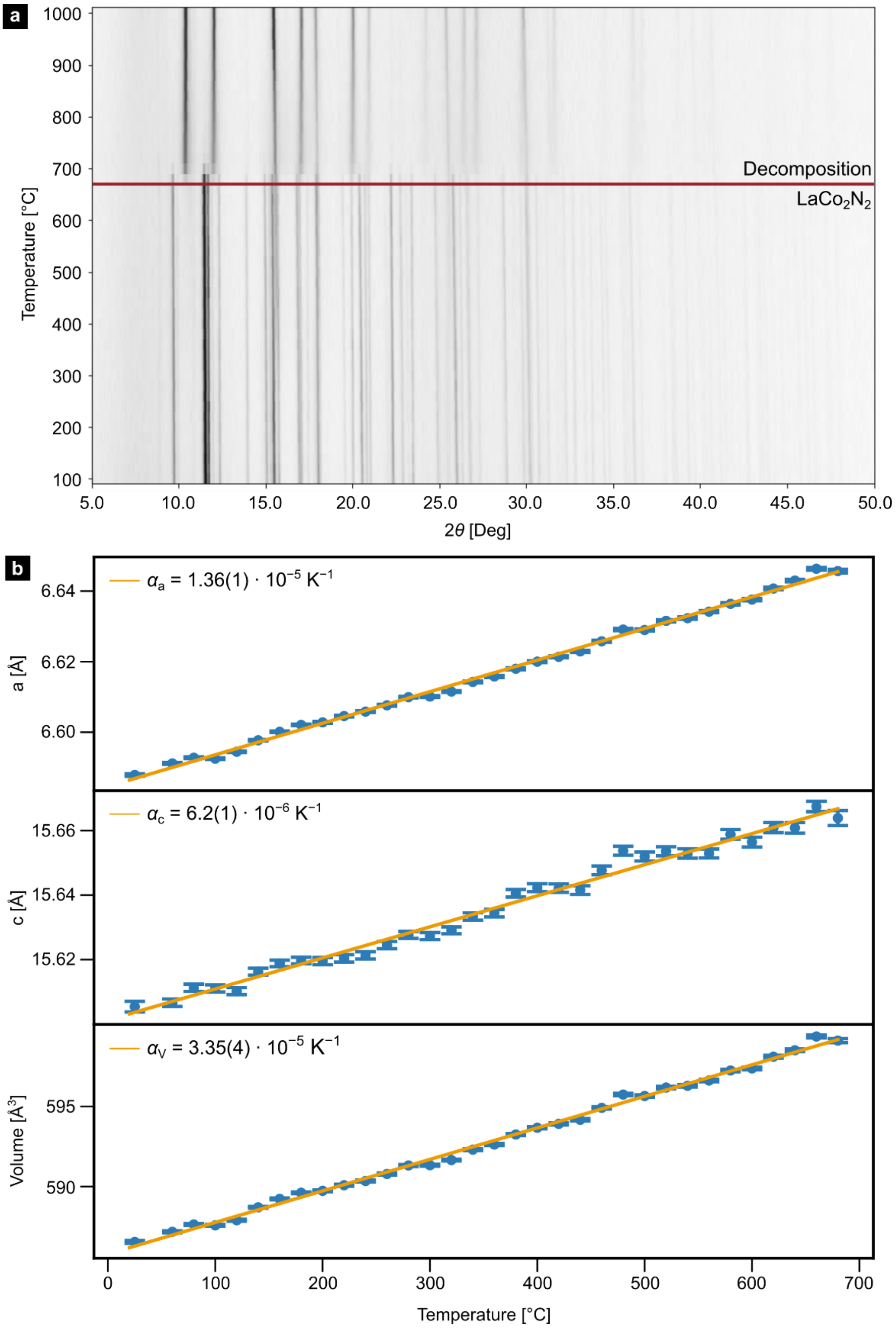

Figure S4: a) Temperature-dependent X-ray powder diffraction of $LaCo_2N_2$. The decomposition is marked with the red line. b) Thermal expansion of the lattice parameters *a*, *c* and the cell volume *V* for $LaCo_2N_2$.

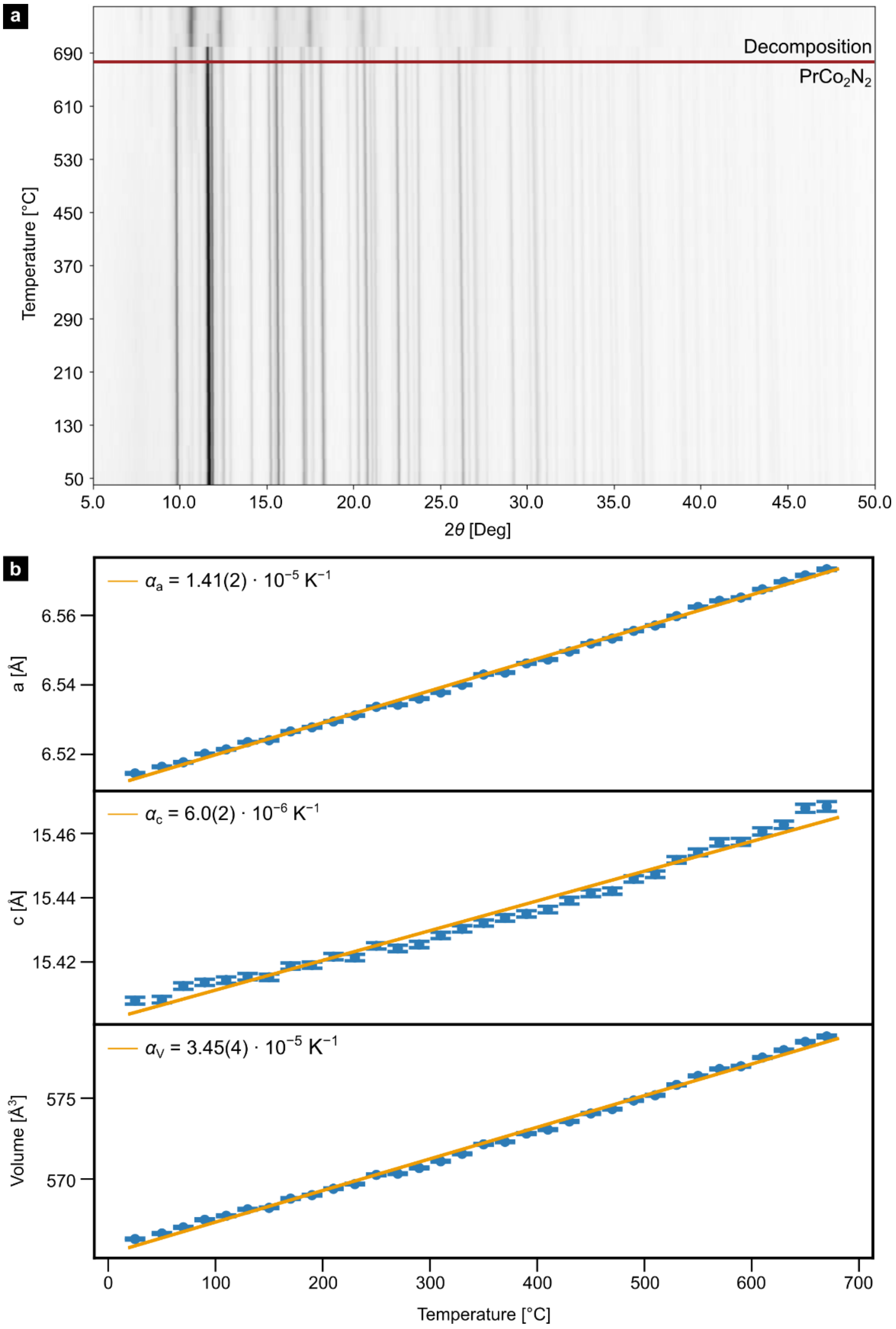

Figure S5: a) Temperature-dependent X-ray powder diffraction of $PrCo_2N_2$. The decomposition is marked with the red line. b) Thermal expansion of the lattice parameters *a*, *c* and the cell volume *V* for $PrCo_2N_2$.

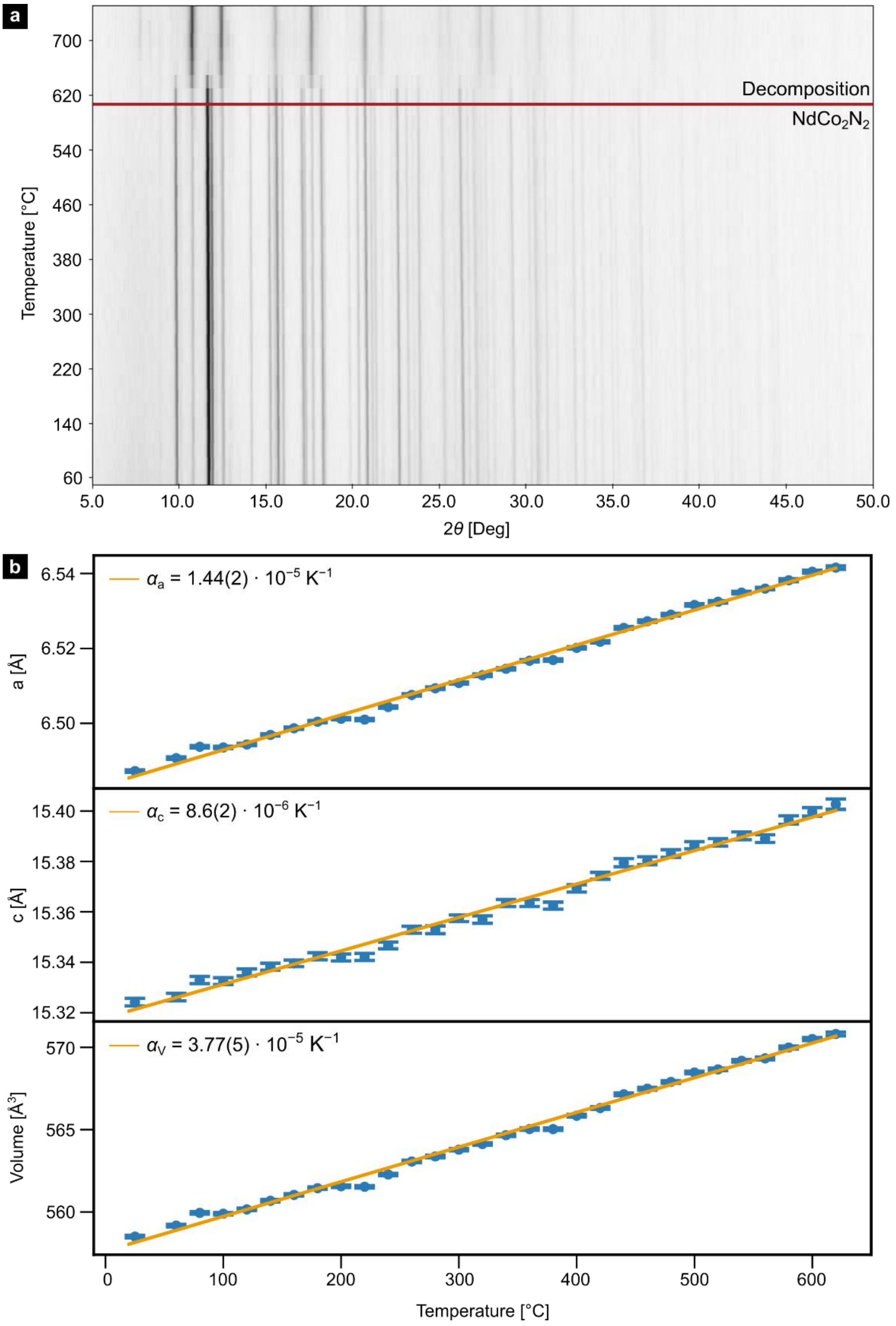

Figure S6: a) Temperature-dependent X-ray powder diffraction of $NdCo_2N_2$. The decomposition is marked with the red line. b) Thermal expansion of the lattice parameters *a*, *c* and the cell volume *V* for $NdCo_2N_2$.

7 References

[1] N. Brese, M. O'keeffe, "Bond-valence parameters for solids", *Acta Crystallographica Section B* 47, no. 2 (1991): 192-197. https://doi.org/10.1107/S0108768190011041.
